# Effects of Low-Level Replacement of Corn with Dried Bamboo Shoot Powder on Growth Performance, Nutrient Utilization, Carcass Traits, Pork Quality, and Colonic Responses in Growing–Finishing Pigs

**DOI:** 10.3390/vetsci13080763

**Published:** 2026-07-30

**Authors:** Rui Tang, Jianfei Zhao, Yong Yang, Jun Yan, Shanchuan Cao, Zhengqun Liu, Jingbo Liu

**Affiliations:** 1 Tianjin Key Laboratory of Animal Molecular Breeding and Biotechnology, Tianjin Engineering Research Center of Animal Healthy Farming, Institute of Animal Science and Veterinary, Tianjin Academy of Agricultural Sciences, Tianjin 300381, China; q1207515167@163.com (R.T.); yjjsxz@163.com (J.Y.); 2 College of Life Sciences and Agri-forestry, Southwest University of Science and Technology, Mianyang 621010, China; zhaojf@swust.edu.cn (J.Z.); yy15681158637@gmail.com (Y.Y.); jingboliu@swust.edu.cn (J.L.); 3 State Key Laboratory of Animal Nutrition and Feeding, Institute of Animal Science, Chinese Academy of Agricultural Sciences, Beijing 100193, China

**Keywords:** dried bamboo shoot powder, corn replacement, growing–finishing pigs, nutrient digestibility, short-chain fatty acids, carcass traits

## Abstract

This study evaluated the effects of low-level replacement of corn with dried bamboo shoot powder (DBSP) on growth performance, nutrient digestibility, serum biochemical, immune, inflammatory, and antioxidant indices, colonic fermentation, microbiota, carcass traits, and meat quality in growing–finishing pigs. A total of 128 healthy pigs (Landrace × Yorkshire × Duroc; initial body weight, 82.90 ± 1.71 kg) were randomly assigned to four dietary treatments: a basal diet (CON) and diets in which 1% (DBS1), 2% (DBS2), or 3% (DBS3) of corn was replaced with DBSP. The experiment included a 5-day adaptation period and a 35-day experimental period. DBSP replacement did not affect growth performance, serum biochemical, immune, inflammatory, or antioxidant indices, or meat quality (*p* > 0.05). Crude protein apparent total tract digestibility increased linearly with DBSP level and was higher in DBS3 than in CON (*p* < 0.05). Colonic acetate concentration increased linearly, and total short-chain fatty acid concentration was affected by dietary treatment. Carcass weight and dressing percentage showed linear responses to increasing DBSP replacement levels. Overall, replacing corn with up to 3% DBSP did not significantly impair growth performance or meat quality and may influence crude protein apparent total tract digestibility, colonic fermentation, and selected carcass traits.

## Simple Summary

Corn is a major energy source in pig diets, but alternative feed ingredients are needed to improve feed-resource use and support more sustainable animal production. Dried bamboo shoot powder is produced from bamboo shoot processing by-products and contains a relatively high amount of fiber, but its value in diets for growing–finishing pigs has not been fully evaluated. In this study, 1%, 2%, or 3% of corn in the diet was replaced with dried bamboo shoot powder. The replacement did not reduce growth performance, blood serum biochemical, immune, inflammatory, and antioxidant indices, or pork quality. It increased crude protein apparent total tract disappearance and was associated with changes in colonic fermentation, especially increased acetate production. Some carcass traits also responded to the replacement level. These findings suggest that dried bamboo shoot powder can be considered as a low-level fibrous alternative ingredient in growing–finishing pig diets.

## 1. Introduction

Corn is one of the most commonly used energy ingredients in diets for growing–finishing pigs. Fluctuations in corn price and competition among feed, food, and industrial uses have increased interest in unconventional feed ingredients for pig production. Food-industry and agro-processing by-products have received increasing attention as alternative resources for livestock diets because they may contribute to feed-resource utilization and circular agricultural production [1]. In addition to growth performance and nutrient utilization, the use of alternative feed ingredients is also related to carcass and meat-quality evaluation, because agro-industrial by-products and former food products can affect product-quality traits depending on their composition and inclusion level [2].

Bamboo shoots are widely consumed as plant-based foods, and their processing generates by-products with potential value for further utilization. Bamboo shoots contain nutrients and bioactive compounds, including protein, vitamins, minerals, carbohydrates, and other plant-derived compounds, and have been recognized as an underutilized plant resource with broader potential in food processing and resource development [3,4]. Dried bamboo shoot powder (DBSP), obtained by drying and grinding bamboo shoot processing by-products, represents a direct form for converting these processing residues into an alternative feed ingredient.

Research on bamboo-derived feed ingredients in pigs has mainly focused on fermented materials. In growing–finishing pigs, fermented bamboo powder has been evaluated primarily as a replacement for wheat bran and has been reported to affect serum biochemical and immune indices, fecal microbial composition, apparent digestibility, carcass traits, and meat quality, with responses depending on inclusion level [5,6]. Bamboo-derived materials have also been examined in young pigs, where fermented bamboo shoot processing waste or fermented bamboo fiber affected growth performance, serum parameters, intestinal barrier-related indices, gut microbiota, and fecal pollutant-related parameters [7,8]. These studies indicate that bamboo-derived materials have been explored in pig nutrition, but most available evidence involves fermented products, wheat bran replacement, or weaned piglets. Information remains limited on the use of unfermented DBSP as a low-level corn-replacing ingredient in diets for growing–finishing pigs.

The present study evaluated the effects and practical applicability of using DBSP, a bamboo shoot processing by-product, to partially replace corn in diets for growing–finishing pigs. Diets were formulated in which 0%, 1%, 2%, or 3% of corn was replaced with DBSP. The objective was to determine the effects of low-level DBSP replacement on growth performance, apparent nutrient digestibility, serum biochemical, immune, inflammatory, and antioxidant indices, colonic short-chain fatty acid concentrations, colonic microbial composition, carcass traits, and pork quality. Because growing–finishing pigs have a relatively mature hindgut microbial ecosystem, colonic short-chain fatty acids and microbial composition were included as supplementary indicators to characterize colonic responses to DBSP replacement.

## 2. Materials and Methods

### 2.1. Animal Ethics Statement

All animal trial protocols were reviewed and approved by the Animal Ethical Review Committee of Southwest University of Science and Technology (Approval No. L2025014; approval date: 12 March 2024). All experimental procedures were conducted in accordance with institutional ethical guidelines.

### 2.2. Animals, Experimental Design and Diets

A total of 128 clinically healthy growing–finishing pigs (64 barrows and 64 gilts; Landrace × Yorkshire × Duroc), with an average initial body weight of 82.90 ± 1.71 kg, were used in this study. Pigs were first allocated to pens according to their initial body weight and sex, with four pigs per pen. The 32 pens were then randomly assigned to one of four dietary treatments, ensuring a balanced distribution of barrows and gilts among treatments, with eight replicate pens per treatment.

Pigs in the control group (CON) were fed the basal diet. In the experimental groups, dried bamboo shoot powder replaced 1%, 2%, or 3% of the corn in the basal diet on a weight-for-weight basis; these treatments were designated DBS1, DBS2, and DBS3, respectively. The DBSP used in this study was produced from bamboo shoot nodes (sections), a processing by-product of *Dendrocalamus latiflorus Munro* collected from Rongchang, Chongqing, China. The bamboo shoot nodes were pretreated, dried in a hot-air oven at 60 °C until the moisture content reached approximately 6%, and subsequently ground and sieved through a 60-mesh screen before being incorporated into the experimental diets. Its concentrations of dry matter, crude protein, ether extract, crude fiber, neutral detergent fiber, acid detergent fiber, ash, calcium, total phosphorus and gross energy are presented in Table 1. The ingredient composition and nutrient levels of the basal diet are shown in Table 2, and the calculated nutrient levels of the basal diet are presented in Table 3. The basal diet was formulated to meet the nutrient requirements for growing–finishing pigs recommended by the NRC (2012). All experimental diets were prepared in mash form throughout the experimental period.

The experiment consisted of a 5-day adaptation period followed by a 35-day experimental period. Throughout the experiment, all pigs had ad libitum access to feed and water, and the ambient temperature was maintained at 18–21 °C. The pigs were housed in pens (2.0 × 3.0 m) equipped with slatted floors, with four pigs per pen. No environmental enrichment was provided during the experimental period. Pens were cleaned regularly, and disinfection and vaccination were performed according to the routine health-management program of the experimental farm. Pig health status was monitored daily, and no mortality or culling occurred during the experimental period.

### 2.3. Apparent Total Tract Digestibility and Chemical Analysis

Apparent total tract digestibility (ATTD) was determined using the indicator method, with chromic oxide (Cr_2_O_3_) as an external marker. From days 28 to 35 of the experimental period, pigs received their respective experimental diets supplemented with 0.5% Cr_2_O_3_. Fresh fecal samples with a visible green color were collected from each pen from days 31 to 35. Fecal samples collected from the same pen were pooled, homogenized, oven-dried, ground, and stored for subsequent chemical analyses.

The concentrations of dry matter, crude protein, ether extract, crude fiber, ash, calcium, and total phosphorus in feed ingredients and diets were determined according to AOAC methods. Gross energy in diets and fecal samples was determined using an adiabatic bomb calorimeter. Chromium concentrations in diets and fecal samples were determined and used for ATTD calculations. The ATTD of each nutrient was calculated as follows:

$$ATTD\;\left(\%\right)=100-100\times \left(\frac{{Cr}_{diet}}{{Cr}_{feces}}\right)\times \left(\frac{{Nutrient}_{feces}}{{Nutrient}_{diet}}\right)$$

where Cr*_diet_* and Cr*_feces_* represent the chromic oxide concentrations in the diet and feces, respectively, and Nutrient*_diet_* and Nutrient*_feces_* represent the concentrations of the corresponding nutrient in the diet and feces, respectively.

### 2.4. Sample Collection

Individual body weights were recorded at 07:00 h after overnight fasting at the beginning and end of the trial. Feed intake was recorded daily for each pen. Average daily gain (ADG), average daily feed intake (ADFI), and feed conversion ratio (FCR) were calculated for each replicate pen. The pen was considered the experimental unit for growth performance.

On day 35 of the experimental period, one pig was randomly selected from each replicate pen for sample collection and subsequent analyses, with jugular blood collection yielding eight serum samples per treatment. Blood samples were allowed to clot at room temperature and then centrifuged to obtain serum. Serum samples were aliquoted and stored at −80 °C until further analysis. Following blood collection, the selected pigs were slaughtered according to standard commercial procedures. Carcass traits were recorded, and longissimus dorsi muscle samples were collected for meat-quality analysis. Colonic digesta samples were collected in sterile cryovials, snap-frozen in liquid nitrogen, and transferred to an ultra-low-temperature freezer at −80 °C for subsequent determination of short-chain fatty acids and gut microbiota analysis.

### 2.5. Serum Biochemical Analysis

Serum biochemical parameters, including alanine aminotransferase (ALT), aspartate aminotransferase (AST), alkaline phosphatase (ALP), total protein (TP), albumin (ALB), glucose (GLU), blood urea nitrogen (BUN), total cholesterol (TC), high-density lipoprotein cholesterol (HDL-C), and low-density lipoprotein cholesterol (LDL-C), were determined using commercial assay kits (Nanjing Jiancheng Bioengineering Institute, Nanjing, China). All procedures were performed according to the manufacturer’s instructions, and absorbance was measured using a microplate reader (Multiskan FC, Thermo Fisher Scientific, Waltham, MA, USA).

### 2.6. Serum Immune and Inflammatory Indices

Serum concentrations of immunoglobulin A (IgA), immunoglobulin G (IgG), immunoglobulin M (IgM), tumor necrosis factor-alpha (TNF-α), interleukin-6 (IL-6), and interleukin-10 (IL-10) were determined using commercial enzyme-linked immunosorbent assay (ELISA) kits according to the manufacturer’s instructions. Absorbance was measured using a microplate reader (Multiskan FC, Thermo Fisher Scientific, Waltham, MA, USA).

### 2.7. Serum Antioxidant Capacity

Serum total antioxidant capacity (T-AOC), superoxide dismutase (SOD), catalase (CAT), glutathione peroxidase (GSH-Px), and malondialdehyde (MDA) were determined using commercial assay kits (Nanjing Jiancheng Bioengineering Institute, Nanjing, China). All procedures and the measurement wavelengths used with the microplate reader (Multiskan FC, Thermo Fisher Scientific, Waltham, MA, USA) followed the manufacturers’ instructions.

### 2.8. Microbiota Profiling Analysis

Total microbial DNA was extracted from colonic digesta using a QIAamp Fast DNA Stool Mini Kit (Qiagen, Hilden, Germany) according to the manufacturer’s instructions. The concentration and purity of extracted DNA were assessed before amplification. The V3–V4 region of the bacterial 16S rRNA gene was amplified using primers 338F (5′-ACTCCTACGGGAGGCAGCAG-3′) and 806R (5′-GGACTACHVGGGTWTCTAAT-3′). Purified amplicons were quantified, pooled in equimolar amounts, and sequenced on an Illumina MiSeq PE300 platform by Majorbio Bio-Pharm Technology Co., Ltd. (Shanghai, China).

Raw sequencing reads were quality-filtered using fastp (version 0.19.6) and merged using FLASH (version 1.2.11). After removal of low-quality sequences and chimeric sequences, the remaining high-quality sequences were clustered into operational taxonomic units (OTUs) at a 97% sequence-similarity threshold using UPARSE software (version 7.1). Low-abundance OTUs (<10 reads in <10% of samples) were removed. Representative sequences from each OTU were taxonomically annotated using the RDP Classifier (version 2.11) against the SILVA 16S rRNA gene database (version 138), with a confidence threshold of 70%.

Alpha diversity was evaluated using the Shannon, ACE, and Chao1 indices. Beta diversity was visualized by principal coordinates analysis, and differences in community structure were assessed using PERMANOVA. Venn diagrams were used to visualize shared and unique OTUs among treatments. Relative abundances were compared at the phylum and genus levels. Differential taxa were identified using the Kruskal–Wallis H test and linear discriminant analysis effect size, with an LDA score threshold of 3.0. Spearman correlation analysis was performed to explore potential associations between differential genera and host phenotypes. These analyses were primarily used for exploratory interpretation of microbiota-related differences and associations.

### 2.9. Carcass Trait Measurements

At the end of the experiment, one pig per pen was randomly selected and slaughtered according to standard commercial procedures. Carcass traits were determined by trained personnel according to the Chinese agricultural industry standard, Technical Specification for Determination of Carcass Traits in Lean-Type Pigs (NY/T 825–2004, Technical Specification for Determination of Carcass Traits in Lean-Type Pigs. Ministry of Agriculture of the People’s Republic of China: Beijing, China, 2004). Measurements included carcass weight, carcass length, dressing percentage, and average backfat thickness. Dressing percentage was calculated as carcass weight divided by preslaughter live body weight. Carcass length was measured, and average backfat thickness was calculated from measurements obtained at the anatomical sites specified by the standard.

### 2.10. Meat Quality Analysis

Longissimus dorsi muscle samples collected between the 10th and 12th ribs on the left side of the carcass were used for meat-quality analysis. Meat-quality measurements were performed by trained personnel according to standardized procedures. All measurements were conducted following predefined protocols to minimize potential bias. Surface fat and connective tissue were removed before analysis. Muscle pH was measured at 45 min and 24 h postmortem using a portable pH meter (HI99163, Hanna Instruments, Woonsocket, RI, USA). Meat color was determined using a colorimeter (CR-400, Konica Minolta, Tokyo, Japan) and expressed as lightness (L*), redness (a*), and yellowness (b*).

Drip loss was determined by weighing fresh muscle samples, suspending them in sealed plastic bags, and reweighing them after storage at 4 °C for 24 h. Drip loss was calculated as the percentage of weight loss relative to the initial sample weight. Cooking loss was determined after samples were heated in a water bath until the internal temperature reached 70 °C, cooled to room temperature, and reweighed. Shear force was measured in cooked samples using a texture analyzer (TA.XTC, Shanghai Bosin Industrial Development Co., Ltd., Shanghai, China). Samples were cut into strips approximately 1.0 cm × 1.0 cm × 3.0 cm and sheared perpendicular to the muscle-fiber direction.

### 2.11. Colonic Short-Chain Fatty Acid Analysis

Colonic concentrations of acetate, propionate, isobutyrate, butyrate, isovalerate, and valerate were determined as previously described, with minor modifications [9]. Briefly, approximately 0.5 g of colonic digesta was thoroughly mixed with 1.5 mL of distilled water. After centrifugation, 1 mL of the supernatant was transferred to a new centrifuge tube, followed by the addition of 200 μL of crotonic acid solution (42 mmol/L) and 200 μL of metaphosphoric acid solution (10%, *w*/*v*). The mixture was vortexed and incubated overnight at 4 °C.

After incubation, samples were centrifuged for 10 min at 4 °C, and the supernatant was collected. An equal volume of diethyl ether was then added, mixed thoroughly, and allowed to stand for 5 min. The diethyl ether layer was carefully collected using a disposable syringe, filtered through a 0.22 μm organic membrane filter, and transferred to amber vials for gas chromatographic analysis. Short-chain fatty acids were analyzed using a gas chromatograph equipped with a flame ionization detector (FID; Agilent 6890 Series GC System, Agilent Technologies, Santa Clara, CA, USA). Separation was performed using a DB-FFAP capillary column (30 m × 0.32 mm × 0.25 μm). Nitrogen was used as the carrier gas. The oven temperature was initially maintained at 60 °C for 2 min, then increased to 150 °C and subsequently to 250 °C. The FID temperature was maintained at 250 °C. Individual SCFA concentrations were calculated from the corresponding standard curves, and total SCFA concentration was calculated as the sum of the six individual SCFAs.

### 2.12. Statistical Analysis

Data were analyzed using SPSS software (version 26.0; IBM Corp., Armonk, NY, USA). Normality and homogeneity of variances were assessed using the Shapiro–Wilk and Levene’s tests, respectively. The pen was considered the experimental unit for growth performance and apparent total tract digestibility. For serum indices, colonic SCFAs, carcass traits, and meat-quality traits, one sampled pig per pen was considered the experimental unit. The overall effect of dietary treatment was evaluated using one-way analysis of variance. When a significant treatment effect was detected, means were compared using Duncan’s multiple-range test. Orthogonal polynomial contrasts were used to assess linear and quadratic responses to increasing levels of corn replacement by dried bamboo shoot powder. Data are presented as means and pooled standard errors of the mean. Differences were considered significant at *p* < 0.05, whereas 0.05 ≤ *p* < 0.10 was considered a tendency.

## 3. Results

### 3.1. Growth Performance and Apparent Nutrient Digestibility

Partial replacement of dietary corn with dried bamboo shoot powder did not significantly affect initial body weight, final body weight, ADG, or ADFI in growing–finishing pigs (Table 4; *p* > 0.05). FCR was not significantly affected by dietary treatment, although tendency-level treatment, linear, and quadratic responses were observed (*p* = 0.098, 0.088, and 0.070, respectively).

The apparent total tract digestibility of crude protein increased linearly with increasing levels of corn replacement by dried bamboo shoot powder (Table 5; *p* < 0.05). Post hoc comparison showed that CP ATTD was higher in the DBS3 group than in the CON, DBS1, and DBS2 groups (*p* < 0.05), whereas no significant differences were observed among CON, DBS1, and DBS2 groups. No significant treatment, linear, or quadratic effects were observed for the apparent total tract digestibility of gross energy, crude fiber, dry matter, or ether extract (*p* > 0.05).

### 3.2. Serum Biochemical, Immune, Inflammatory, and Antioxidant Indices

As shown in Table 6, partial replacement of corn with dried bamboo shoot powder had no significant effects on serum biochemical indices in growing–finishing pigs (*p* > 0.05). No significant linear or quadratic responses were observed with increasing levels of corn replacement by dried bamboo shoot powder (*p* > 0.05).

Serum immune and inflammatory indices are presented in Table 7. Partial replacement of corn with dried bamboo shoot powder had no significant effects on serum IgA, IgG, IgM, TNF-α, IL-6, or IL-10 concentrations (*p* > 0.05). No significant linear or quadratic responses were observed for these indices (*p* > 0.05).

As shown in Table 8, partial replacement of corn with dried bamboo shoot powder had no significant effects on serum T-AOC, SOD, CAT, GSH-Px, or MDA levels (*p* > 0.05). However, serum SOD activity showed a linear decreasing tendency as the level of corn replacement by dried bamboo shoot powder increased (*p* = 0.062).

### 3.3. Colonic Short-Chain Fatty Acids

Colonic SCFA concentrations are presented in Table 9. Partial replacement of corn with dried bamboo shoot powder tended to affect colonic acetate concentration (*p* = 0.075), which increased linearly with increasing levels of corn replacement (*p* < 0.05). Dietary treatment significantly affected total SCFA concentration (*p* < 0.05), with a linear increasing tendency as the level of corn replacement increased (*p* = 0.057). However, propionate, isobutyrate, butyrate, isovalerate, and valerate concentrations were not significantly affected by dietary treatment (*p* > 0.05).

### 3.4. Colonic Microbiota Diversity

**Figure 1:**
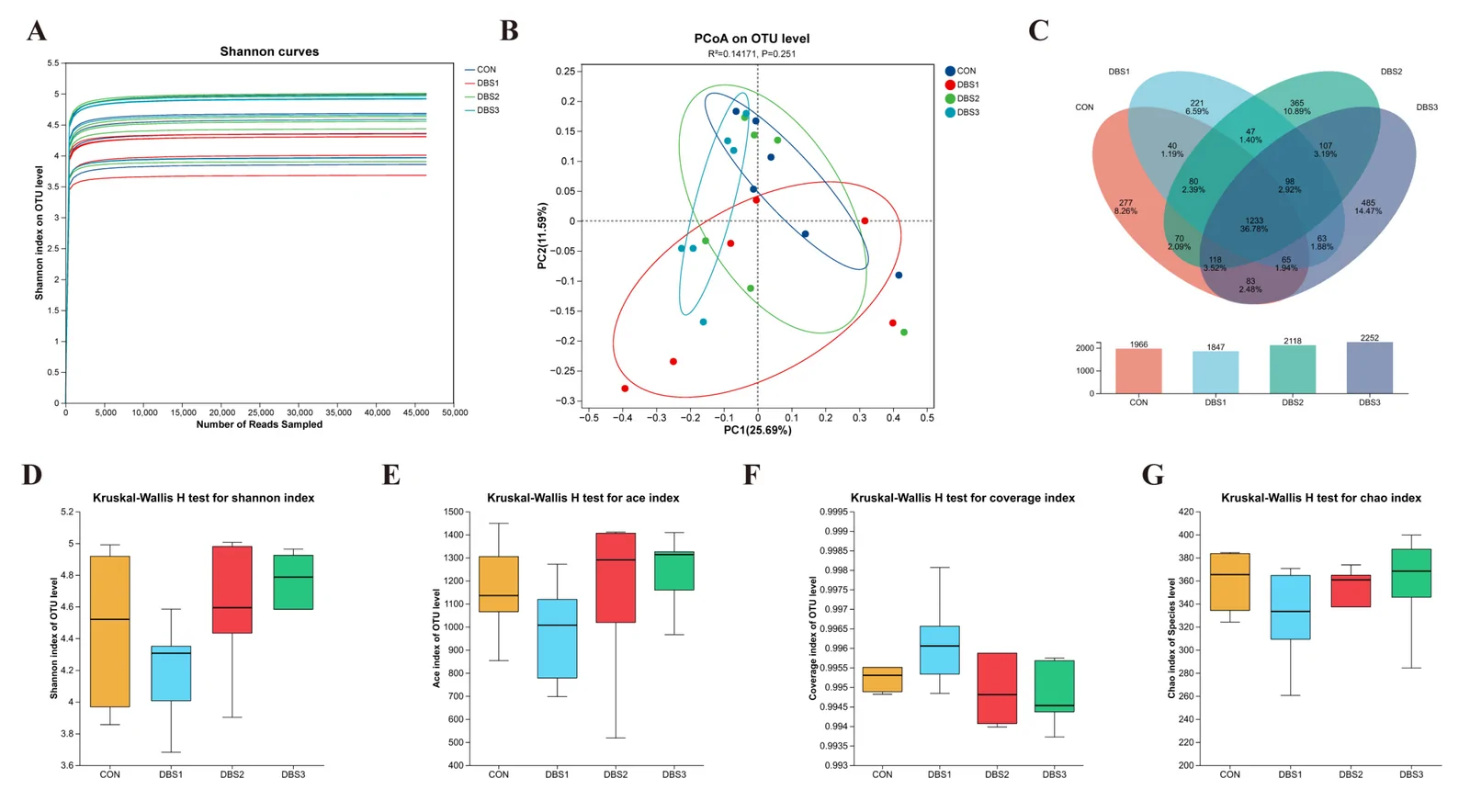
Effects of partial replacement of corn with dried bamboo shoot powder on colonic microbial diversity and OTU distribution in growing–finishing pigs. (**A**) Shannon rarefaction curves of colonic microbiota showing the relationship between sequencing depth and microbial diversity. The curves approached plateaus with increasing numbers of reads, indicating sufficient sequencing depth. (**B**) Principal coordinates analysis (PCoA) of colonic microbiota at the OTU level. Each point represents one sample; the R^2^ and *p* values were obtained from PERMANOVA. (**C**) Venn diagram showing shared and unique OTUs among dietary treatments; the bar chart indicates the total number of OTUs detected in each group. (**D**–**G**) Alpha-diversity indices, including Shannon (**D**), ACE (**E**), Good’s coverage (**F**), and Chao1 (**G**). CON, basal diet; DBS1, DBS2, and DBS3, diets in which 1%, 2%, and 3% of corn, respectively, were replaced with dried bamboo shoot powder. In box plots, the box represents the interquartile range, the horizontal line inside the box indicates the median, and whiskers indicate the data distribution range.

16S rRNA gene sequencing was used to evaluate the effects of partial replacement of corn with DBSP on the colonic microbiota of growing–finishing pigs. After quality filtering, paired-end merging, and removal of low-quality sequences, a total of 2,043,557 high-quality sequences were obtained, with an average of approximately 68,119 sequences per sample. Rarefaction curves based on the Shannon index gradually approached plateaus with increasing sequencing depth, indicating that the sequencing depth was sufficient to capture the microbial diversity of colonic samples (Figure 1A). Good’s coverage values exceeded 99% across all dietary treatments, further confirming adequate sequencing coverage (Figure 1F). At the OTU level, PCoA showed sample-level variation among dietary treatments (Figure 1B). However, PERMANOVA revealed no significant difference in the overall microbial community structure among groups (*R*^2^ = 0.1417, *p* = 0.251).

The Venn diagram showed that 1233 OTUs were shared among the four dietary treatments, accounting for 36.78% of all detected OTUs (Figure 1C). The numbers of unique OTUs were 277, 221, 365, and 485 in the CON, DBS1, DBS2, and DBS3 groups, respectively. Alpha-diversity analysis showed that partial replacement of corn with DBSP had no significant effects on the Chao1, Shannon, or ACE indices (Figure 1D,E,G).

### 3.5. Colonic Microbiota Composition and Differential Taxa

**Figure 2:**
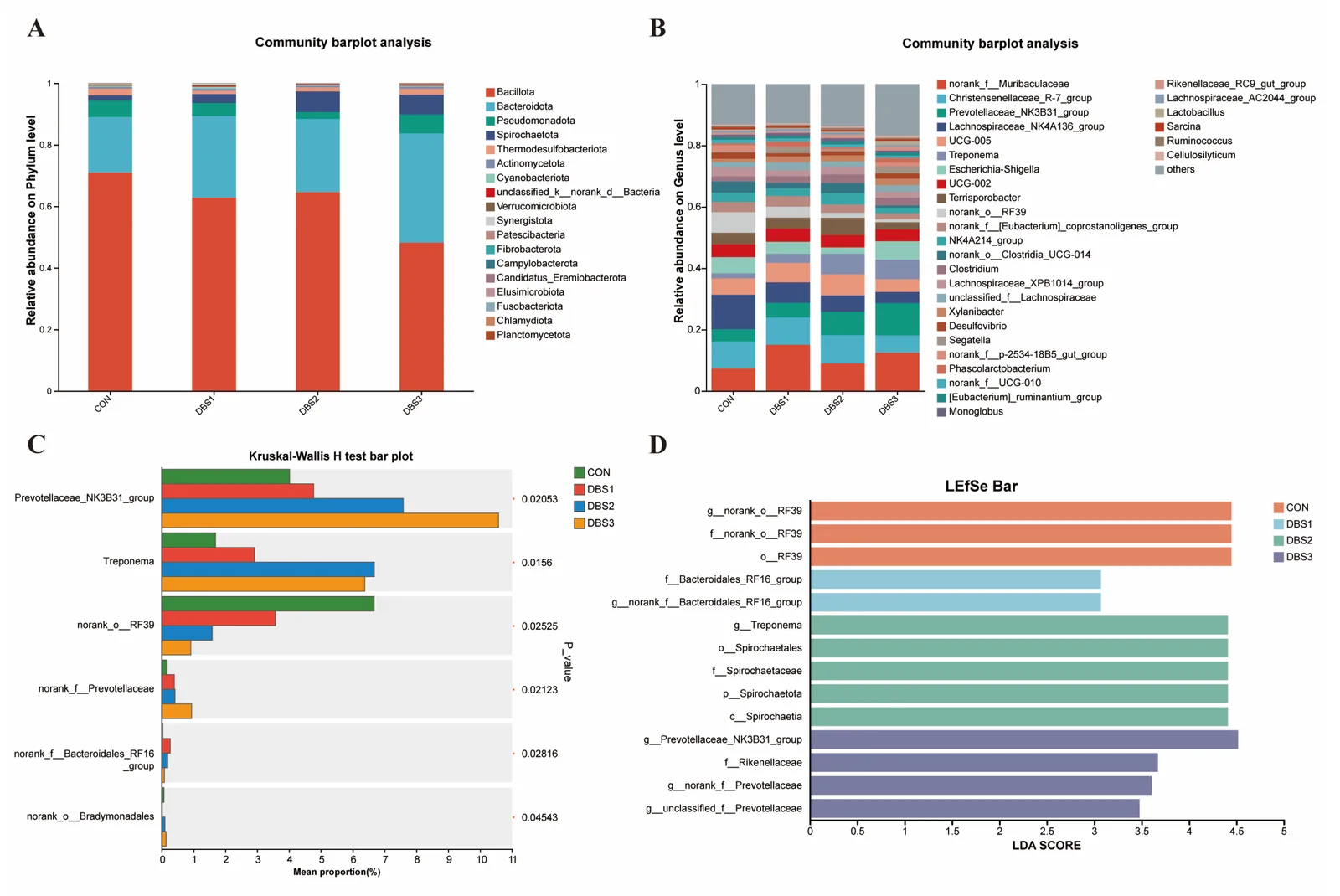
Effects of partial replacement of corn with dried bamboo shoot powder on the colonic microbial composition of growing–finishing pigs. (**A**) Relative abundances of the predominant bacterial phyla. (**B**) Relative abundances of the predominant bacterial genera. (**C**) Differential genera among dietary treatments identified by the Kruskal–Wallis H test. (**D**) Differential taxa identified by linear discriminant analysis effect size (LEfSe), with an LDA score threshold of 3.0. CON, basal diet; DBS1, DBS2, and DBS3, diets in which 1%, 2%, and 3% of corn, respectively, were replaced with dried bamboo shoot powder.

To characterize the taxonomic composition of colonic microbial communities, relative abundances were compared at the phylum and genus levels. At the phylum level, *Bacillota* and *Bacteroidota* were the predominant phyla in all groups. The relative abundance distributions of several dominant phyla, including *Bacillota*, *Bacteroidota*, and *Spirochaetota*, varied among dietary treatments (Figure 2A).

At the genus level, the predominant taxa included *norank_f_Muribaculaceae*, *Christensenellaceae_R-7_group*, *Prevotellaceae_NK3B31_group*, *Lachnospiraceae_NK4A136_group*, *Treponema*, *Escherichia-Shigella*, *UCG-005*, *UCG-002*, and *Terrisporobacter* (Figure 2B). The Kruskal–Wallis H test identified significant differences in the relative abundances of *Prevotellaceae_NK3B31_group*, *Treponema*, *norank_o_RF39*, *norank_f_Prevotellaceae*, *norank_f_Bacteroidales_RF16_group*, and *norank_o_Bradymonadales* among treatments (Figure 2C). Specifically, *Prevotellaceae_NK3B31_group* was more abundant in the DBS3 group, *Treponema* was more abundant in the DBS2 and DBS3 groups, and *norank_o_RF39* was primarily enriched in the CON group.

LEfSe analysis further identified discriminative taxa among the dietary treatments. The DBS3 group was characterized by enrichment of *Prevotellaceae_NK3B31_group*, *Rikenellaceae*, *norank_f_Prevotellaceae*, and *unclassified_f_Prevotellaceae*. The DBS2 group was characterized by *Treponema* and *Spirochaetota*-related taxa, whereas the DBS1 group was characterized by *Bacteroidales_RF16_group*-related taxa. *RF39*-related unclassified taxa were enriched in the CON group (Figure 2D).

### 3.6. Carcass Traits and Pork Quality

Carcass traits were partly affected by dried bamboo shoot powder replacement (Table 10). Partial replacement of corn with dried bamboo shoot powder had no significant overall effects on carcass length or average backfat thickness (*p* > 0.05). However, carcass weight increased linearly with increasing levels of corn replacement by dried bamboo shoot powder (*p* = 0.027). Dressing percentage tended to be affected by dietary treatment (*p* = 0.096) and increased linearly as the replacement level increased (*p* = 0.042). In addition, average backfat thickness showed a linear decreasing tendency (*p* = 0.083).

Meat-quality traits were not significantly affected by dietary treatment (Table 11). Partial replacement of corn with dried bamboo shoot powder had no significant effects on muscle pH at 45 min or 24 h postmortem, meat color, drip loss, cooking loss, or shear force (*p* > 0.05). No significant linear or quadratic responses were observed for these traits (*p* > 0.05).

### 3.7. Associations Between Key Bacteria and Host Phenotypes

**Figure 3:**
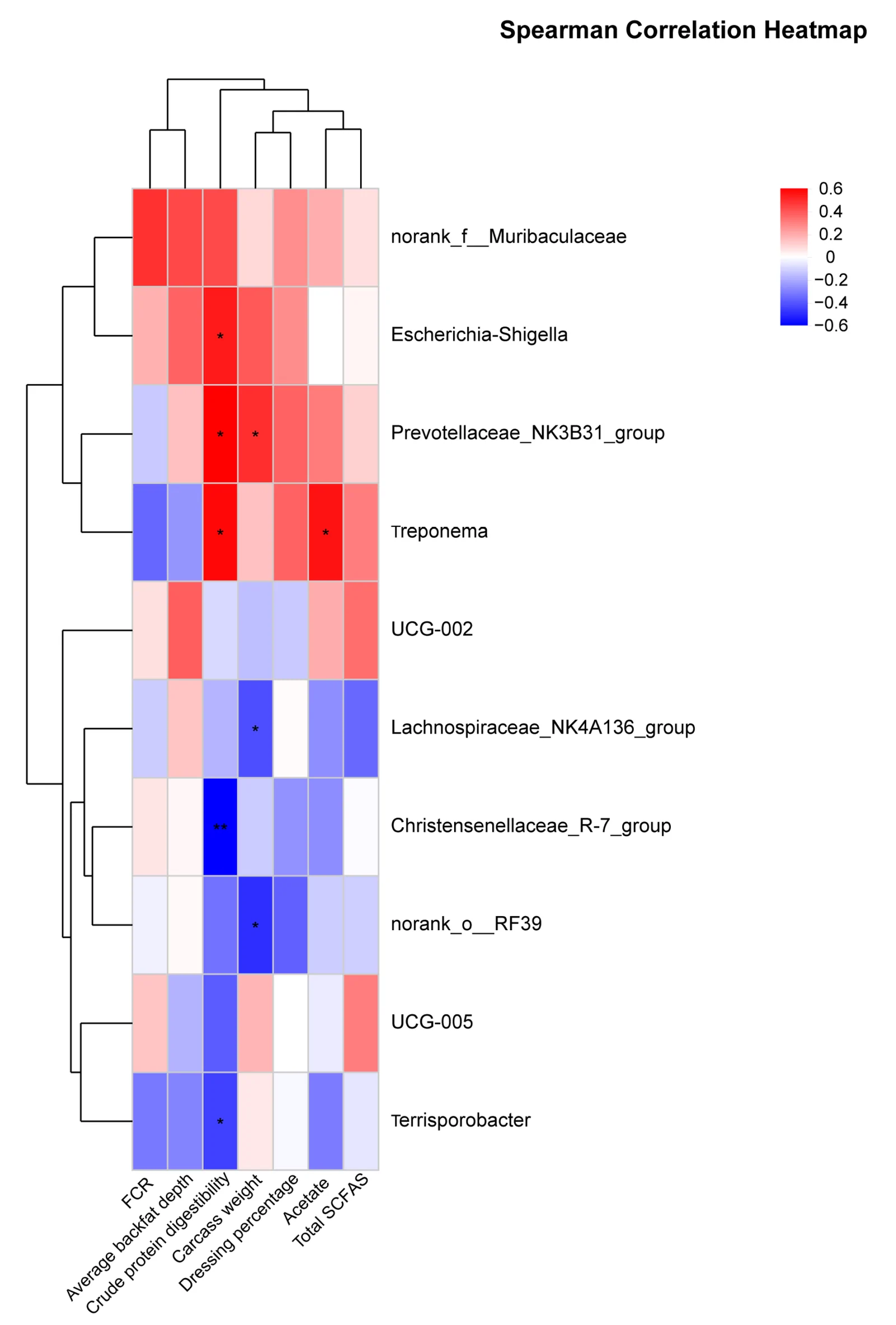
Spearman correlation heatmap between differential colonic bacterial genera and host phenotypic parameters in growing–finishing pigs. Red indicates positive correlations, whereas blue indicates negative correlations. The color intensity represents the Spearman correlation coefficient. Asterisks indicate significant correlations (* *p* < 0.05, ** *p* < 0.01). FCR, feed conversion ratio; SCFA, short-chain fatty acid.

Spearman correlation analysis was performed to evaluate associations between key differential genera and host phenotypic traits, and the results are shown in Figure 3. Several differential genera were significantly associated with the apparent total tract digestibility of crude protein, acetate and total short-chain fatty acid concentrations, carcass weight, dressing percentage, average backfat thickness, and feed conversion ratio. Specifically, *Escherichia-Shigella* was positively correlated with crude protein digestibility (*p* < 0.05). *Prevotellaceae_NK3B31_group* was positively correlated with crude protein digestibility and dressing percentage (*p* < 0.05). *Treponema* was positively correlated with crude protein digestibility and acetate concentration (*p* < 0.05). In contrast, *Christensenellaceae_R-7_group* was negatively correlated with crude protein digestibility (*p* < 0.01). In addition, *Lachnospiraceae_NK4A136_group* and *norank_o_RF39* were negatively correlated with dressing percentage (*p* < 0.05), whereas *Terrisporobacter* was negatively correlated with crude protein digestibility (*p* < 0.05).

## 4. Discussion

In the present study, low-level replacement of dietary corn with DBSP did not significantly affect ADG, ADFI, or FCR in growing–finishing pigs, indicating that the 1–3% replacement levels did not compromise production performance during the 35-day experimental period. The replacement levels used in this study were selected as an initial evaluation strategy to assess the feasibility of incorporating DBSP into growing–finishing pig diets. Since DBSP is derived from bamboo shoot processing by-products and differs from conventional cereal ingredients in nutrient composition and substrate characteristics, relatively low replacement levels were adopted to minimize potential effects associated with differences in fiber characteristics, nutrient balance, and feed acceptance. Corn is primarily used as an energy ingredient in growing–finishing pig diets, whereas DBSP is derived from bamboo shoot processing by-products and differs from conventional cereal ingredients in nutrient composition and substrate characteristics. Bamboo shoots contain abundant dietary fiber and various bioactive compounds, while some potential anti-nutritional components, such as cyanogenic glycosides, have also been reported depending on bamboo species and processing conditions [3]. Processing methods, including drying and other preservation treatments, may influence the levels and availability of these compounds. In the present study, DBSP was subjected to drying and grinding before dietary incorporation; however, the specific bioactive compounds and anti-nutritional factors in DBSP were not directly determined. Therefore, the growth response to DBSP replacement likely depends on the replacement level, nutrient balance of the complete diet, feed acceptance, and the capacity of growing–finishing pigs to adapt to changes in dietary composition. In the present study, DBSP was included at low levels and replaced corn on an equal weight basis within nutritionally formulated diets, which may have reduced the direct effect of ingredient-composition differences on growth performance. The tendency-level response in FCR should not be interpreted as an improvement in feed efficiency. Compared with growth performance, CP ATTD, colonic acetate concentration, selected colonic bacterial taxa, and several carcass traits showed more evident responses to DBSP replacement, suggesting that the responses to low-level ingredient replacement were more evident in nutrient utilization and the colonic environment than in growth rate.

Comparable responses have been reported in studies evaluating corn replacement or alternative ingredients in growing–finishing pig diets. Studies using ground brown rice or rice as corn-replacing ingredients in growing–finishing pig diets have shown that growth performance can remain stable when the complete diet is nutritionally adequate, whereas dietary responses may be more evident in nutrient digestibility, intestinal microbiota, metabolites, or pork-quality traits [10,11]. Compared with cereal ingredients such as ground brown rice or rice, DBSP differs more markedly from corn in nutritional properties. Thus, the stable growth performance observed in the present study is more likely related to the low replacement levels and formulation control of the complete diet, rather than to nutritional equivalence between DBSP and corn. Bamboo-derived ingredients have also been examined in growing–finishing pig diets, particularly in studies using fermented bamboo powder as a replacement for wheat bran [5,6]. However, fermentation can alter the chemical composition, palatability, and microbial substrate availability of bamboo-derived materials. In addition, wheat bran and corn differ in their nutritional functions within complete diets. Therefore, growth responses among bamboo-derived ingredient studies should be compared in relation to processing method, replaced ingredient, and inclusion level.

The increase in CP ATTD represented one of the main digestive responses observed in this study. CP ATTD showed a significant linear response with increasing DBSP replacement level; however, this pattern was mainly attributed to the higher value observed in DBS3, because CON, DBS1, and DBS2 showed comparable values. Therefore, the observed response may represent a more evident effect at the highest inclusion level rather than a uniform increase across all replacement levels. This result indicates that apparent protein disappearance across the total gastrointestinal tract was maintained and, at the 3% replacement level, increased. However, ATTD cannot distinguish small-intestinal digestion and absorption from hindgut microbial metabolism; therefore, the increase in CP ATTD should not be directly interpreted as enhanced amino acid absorption or protein deposition. Protein digestibility measured at the total-tract level can be influenced by diet composition, digestive processes, and nitrogen excretion patterns, particularly when dietary protein supply and fermentable substrates alter microbial nitrogen metabolism in the hindgut [12,13]. Based on these findings, the higher CP ATTD observed in the present study may reflect changes in apparent nitrogen disappearance and fecal nitrogen output across the total digestive tract, rather than a simple improvement in small-intestinal protein digestion.

The distinction between ATTD and ileal digestibility is also important for explaining why the increase in CP ATTD was not accompanied by significant improvements in ADG or FCR. Amino acids are mainly absorbed before the terminal ileum, whereas hindgut microorganisms can utilize nitrogenous substrates and form microbial biomass, thereby altering fecal nitrogen output and affecting total-tract digestibility measurements. For estimating amino acid availability in pig feed ingredients, ileal digestibility is generally preferred over total-tract digestibility because it avoids the confounding influence of hindgut microbial metabolism [14]. Therefore, the higher CP ATTD in DBS3 should be interpreted as an improvement in an apparent digestibility index rather than direct evidence of increased amino acid availability. Further measurements of standardized ileal amino acid digestibility and nitrogen metabolism would be required to determine whether DBSP affects protein and amino acid utilization at the ileal or metabolic level.

The absence of significant changes in serum biochemical indices, immunoglobulin concentrations, inflammatory cytokines, and most antioxidant indices indicates that low-level DBSP replacement did not impose an evident systemic physiological burden. Although SOD activity showed a linear decreasing tendency, MDA did not increase, and T-AOC, CAT, and GSH-Px were not significantly altered. Therefore, this isolated tendency in SOD should not be interpreted as evidence of oxidative stress. Dietary responses in serum indices and microbial composition are often dependent on animal stage, ingredient characteristics, and the overall dietary context, rather than on a single compositional feature of the replacement ingredient [15,16]. In the present study, the overall serum results indicate that replacing corn with 1–3% DBSP did not induce obvious adverse metabolic, immune, inflammatory, or oxidative stress responses.

SCFAs are major microbial metabolites produced from substrates reaching the hindgut and can reflect changes in diet–microbiota metabolic interactions [17]. Different substrates entering the hindgut may generate distinct SCFA profiles depending on their chemical structure, microbial accessibility, and fermentation pathways [18]. In the present study, colonic acetate concentration increased linearly with increasing DBSP replacement level, although the overall treatment effect on acetate only reached a tendency level. Total SCFA concentration was affected by dietary treatment and showed a linear increasing tendency. In contrast, propionate, butyrate, and branched-chain fatty acids were not significantly affected. These results indicate that the SCFA response associated with DBSP replacement was mainly characterized by increased acetate production rather than a general increase in all SCFA components.

The increase in acetate may be related to changes in the substrate profile entering the colon after DBSP replacement of corn. Substrate utilization in the porcine hindgut is influenced by substrate structure, solubility, fermentability, and passage rate, and cannot be predicted solely from soluble/insoluble classification or total fiber content [18,19]. Based on this concept, the increase in acetate in the present study does not directly demonstrate enhanced degradation of a specific fiber fraction. Rather, it may reflect altered availability of colonic substrates after low-level replacement of corn with DBSP. The absence of significant changes in branched-chain fatty acids suggests that DBSP replacement did not markedly increase colonic protein putrefaction. Increased fermentable protein reaching the distal intestine can enhance microbial amino acid catabolism and the production of related metabolites [20]. In this context, the stable branched-chain fatty acid profile observed here indicates that the colonic response to DBSP was mainly acetate-related rather than a broad increase in fermentation or protein catabolism.

The colonic microbiota results further support the interpretation of a localized taxonomic response rather than a global restructuring of the microbial ecosystem. PCoA and PERMANOVA did not show significant separation of colonic microbial communities among treatments, and Chao1, Shannon, ACE, and coverage indices were not significantly affected. These results indicate that replacing 1–3% of dietary corn with DBSP did not markedly alter colonic microbial α-diversity or overall β-diversity. The response of the porcine gut microbiota to dietary composition depends on animal stage, dietary background, ingredient characteristics, and the magnitude of dietary intervention [15,16]. Given the low replacement levels used in the present study and the relatively stable gut microbial ecosystem of growing–finishing pigs, the absence of a major shift in overall microbial structure may be related to the low replacement levels and the relatively stable gut microbial ecosystem of growing–finishing pigs.

Further taxonomic analysis showed that *Prevotellaceae_NK3B31_group*, *Treponema*, and related taxa differed among treatments. *Prevotellaceae*- and *Treponema*-related taxa have been associated with the utilization of plant-derived substrates and complex carbohydrates, although their functional roles vary depending on host stage, intestinal site, and dietary background. The role of *Prevotella*-related taxa in pigs is context-dependent and may be associated with production performance, feed utilization, and host health depending on diet and physiological state [21]. Links between intestinal microbial composition, feed efficiency, and substrate utilization have been described in pigs, including metagenomic evidence that animals differing in apparent fiber digestibility may harbor distinct microbial and functional gene profiles [22,23]. In the present study, *Prevotellaceae*-related taxa were mainly enriched in DBS3, whereas *Treponema* was more abundant in DBS2 and DBS3; LEfSe analysis further indicated that *Treponema*- and *Spirochaetota*-related taxa were mainly enriched in DBS2. These changes are directionally consistent with the altered substrate profile caused by DBSP replacement and the increase in colonic acetate concentration. However, this consistency only indicates that these taxa may be associated with DBSP- related colonic responses and does not directly demonstrate specific substrate-degrading functions.

Correlation analysis provided additional information on the potential associations between bacterial taxa and host phenotypes. However, considering the exploratory nature of these correlation analyses and the potential risk of false-positive associations arising from multiple comparisons, these findings should be interpreted cautiously and require further validation rather than being considered as causal evidence. *Prevotellaceae_NK3B31_group* and *Treponema* were positively correlated with CP ATTD, dressing percentage, or colonic acetate concentration, whereas *Christensenellaceae_R-7_group*, *Terrisporobacter*, *Lachnospiraceae_NK4A136_group*, and *norank_o_RF39* were negatively correlated with selected host traits. Complex associations between gut microbiota, nutrient utilization, feed efficiency, and production traits have been reported in pigs, but such associations are generally context-dependent and do not establish causality [24,25]. In the present study, *Escherichia-Shigella* was also positively correlated with CP ATTD; however, inflammatory indices and growth performance did not show adverse changes, so this association should be interpreted cautiously and should not be used to infer a beneficial role. Functional inference based only on 16S rRNA gene sequencing is limited because this approach primarily provides taxonomic rather than direct functional information [26]. In addition, although OTU-based clustering at a 97% sequence similarity threshold remains widely used in microbial ecology studies, it may provide lower taxonomic resolution than ASV-based approaches, such as DADA2 or Deblur. Accordingly, the differential taxa and correlation results in the present study should be considered as a basis for future functional verification rather than as mechanistic conclusions. Metagenomic, metatranscriptomic, or targeted metabolomic analyses would be needed to determine whether these taxa directly participate in nitrogen metabolism, acetate production, or other substrate-utilization processes.

Carcass traits showed limited but measurable responses to DBSP replacement. Carcass weight increased linearly with increasing DBSP replacement level, dressing percentage showed a tendency for a treatment effect and increased linearly, and average backfat thickness tended to decrease linearly. Because ADG and FCR were not significantly changed, these carcass responses should not be interpreted as evidence of improved overall production efficiency. Feed efficiency and carcass output in pigs are integrated outcomes of maintenance requirements, lean tissue deposition, fat deposition, and nutrient partitioning, which makes isolated carcass responses difficult to interpret as direct evidence of improved production efficiency [27]. Studies using plant-derived or agro-industrial by-product ingredients in finishing pig diets further indicate that carcass and meat-quality responses depend on ingredient composition and inclusion level [28,29]. Based on the present results, the carcass responses to DBSP are more appropriately interpreted as minor changes in carcass output or nutrient partitioning rather than clear advantages in weight gain or feed efficiency.

Meat-quality traits were relatively stable. DBSP replacement did not significantly affect muscle pH, meat color, drip loss, cooking loss, or shear force, indicating that 1–3% DBSP replacement did not adversely affect postmortem glycolysis, water-holding capacity, tenderness, or visual meat quality under the present dietary conditions. The effects of alternative feed ingredients on red meat quality are generally ingredient-specific and inclusion-level dependent, particularly when agro-industrial by-products or former food products are used in livestock diets [2]. Evidence from studies using palm kernel meal or palm kernel expellers as corn-replacing or unconventional ingredients further supports the need to evaluate pork quality when defining the application boundaries of plant-processing by-products in pig diets [30,31,32]. Compared with the linear or tendency-level responses observed in individual carcass traits, the absence of adverse effects on pork quality provides a more direct production-related outcome.

Overall, low-level replacement of corn with unfermented DBSP did not impair growth performance, serum physiological status, or pork quality in growing–finishing pigs. The main responses were increased CP ATTD, a selective increase in colonic acetate concentration, changes in selected colonic bacterial taxa, and linear responses in several carcass traits. These results are more appropriately characterized as limited digestive and colonic responses to low-level DBSP replacement rather than as evidence of improved growth performance or a defined fiber-degradation mechanism. This study has several limitations. First, the replacement levels were low, and the findings should not be extrapolated to higher DBSP inclusion levels. Second, the experimental period was relatively short, and longer-term studies across broader body-weight ranges are needed to determine the practical feeding value of DBSP throughout the growing–finishing period. Third, digestibility was evaluated as ATTD, which cannot replace ileal amino acid digestibility. Fourth, 16S rRNA gene sequencing cannot directly demonstrate microbial function. Finally, energy utilization, responses under different formulation conditions, and the economic value of DBSP were not evaluated. Future studies integrating standardized ileal amino acid digestibility, energy metabolism, metagenomics, targeted metabolomics, and cost–benefit analyses under commercial production conditions would help clarify the nutritional value, functional responses, economic feasibility, and practical application range of DBSP under different replacement levels and dietary backgrounds.

## 5. Conclusions

Within the conditions of this study, replacing 1% to 3% of dietary corn with dried bamboo shoot powder did not impair growth performance, serum biochemical, immune, inflammatory or antioxidant indices, or pork quality in growing–finishing pigs. The 3% replacement level increased the apparent total tract digestibility of crude protein, and increasing replacement levels were associated with a linear increase in colonic acetate concentration and modest linear responses in carcass weight and dressing percentage. Microbiota analysis indicated that DBSP replacement was associated with changes in selected colonic bacterial taxa, whereas overall colonic microbial diversity and community structure were not markedly altered. These findings suggest that dried bamboo shoot powder can be considered as a low-level corn-replacing alternative feed ingredient in diets for growing–finishing pigs, although further studies are needed to evaluate its amino acid digestibility, energy utilization, microbial functions, and economic value.

## Figures and Tables

**Table 1:** Nutrient composition of dried bamboo shoot powder (air-dry basis).

Item	Content
Dry matter (%)	92.5
Crude protein (%)	9.67
Ether extract (%)	2.39
Crude fiber (%)	28.68
Neutral detergent fiber (%)	49.72
Acid detergent fiber (%)	33.46
Ash (%)	4.03
Total Ca (%)	0.78
Total P (%)	0.24
Gross energy (kcal/kg)	3978

**Table 2:** Ingredient composition of experimental diets (% as-fed basis).

Item	CON	DBSP1	DBSP2	DBSP3
Corn	80.90	79.90	78.90	77.90
DBSP	0	1.00	2.00	3.00
Soybean meal	11.00	11.00	11.00	11.00
Wheat bran	4.00	4.00	4.00	4.00
Soybean oil	1.00	1.00	1.00	1.00
Dicalcium phosphate	1.00	1.00	1.00	1.00
Limestone	0.60	0.60	0.60	0.60
Salt	0.40	0.40	0.40	0.40
L-Lys HCL (78.8%)	0.40	0.40	0.40	0.40
DL-Met (98%)	0.10	0.10	0.10	0.10
L-Thr (97.5%)	0.20	0.20	0.20	0.20
Choline chloride	0.10	0.10	0.10	0.10
Premix ^1^	0.30	0.30	0.30	0.30
Total	100.00	100.00	100.00	100.00

Note. DBSP = dried bamboo shoot powder. DBSP1, DBSP2, and DBSP3 indicate diets in which 1%, 2%, and 3% of corn were replaced with DBSP, respectively. ^1^ Per kilogram of diet, the premix provided vitamins and trace minerals as follows: VA_5_ 512 IU; VD_2_ 250 IU; VE 24 mg; VK 3 mg; VB_12_ 0.024 mg; Riboflavin 6 mg; D-pantothenic acid 15 mg; Niacin 20 mg; VB_6_ 3 mg; biotin 0.15 mg; folic acid 1.2 mg; Fe 100 mg; Mn 30 mg; Cu 15 mg; I 0.3 mg; Se 0.2 mg; and Zn 100 mg.

**Table 3:** Calculated nutrient levels of the basal diet.

Nutrient %	Basal Diet
Digestible energy, kcal/kg	3407
Crude protein	12.42
Ca	0.54
P	0.50
Ca: P	1.08

**Table 4:** Effects of partial replacement of corn with dried bamboo shoot powder on growth performance in growing–finishing pigs.

Item	DBSP Replacement Level (% of Diet)				SEM	*p*-Value		
	0 (CON)	1.00	2.00	3.00		Treatment	Linear	Quadratic
Initial BW, kg	82.59	83.26	82.41	83.32	0.62	0.648	0.633	0.846
Final BW, kg	121.43	120.20	121.04	121.65	1.15	0.817	0.774	0.427
ADG, kg/day	1.11	1.06	1.11	1.09	0.02	0.159	0.988	0.241
ADFI, kg/day	3.14	3.02	3.15	2.97	0.09	0.421	0.355	0.741
FCR, kg feed/kg weight gain	2.83	2.86	2.86	2.71	0.05	0.098	0.088	0.070

Note. *n* = 8. SEM = standard error of mean. CON = control diet; DBSP = dried bamboo shoot powder. BW = body weight; ADG = average daily gain; ADFI = average daily feed intake; FCR = feed conversion ratio.

**Table 5:** Effects of partial replacement of corn with dried bamboo shoot powder on apparent nutrient digestibility in growing–finishing pigs.

Item	DBSP Replacement Level (% of Diet)				SEM	*p*-Value		
	0 (CON)	1.00	2.00	3.00		Treatment	Linear	Quadratic
Gross energy, %	82.36	83.05	82.12	82.35	0.63	0.746	0.731	0.724
Crude protein, %	69.98 ^b^	69.52 ^b^	70.31 ^b^	73.77 ^a^	1.09	0.039	0.019	0.082
Crude fiber, %	43.87	45.80	45.68	45.86	1.05	0.483	0.221	0.408
Dry matter, %	82.53	82.30	82.41	81.87	0.39	0.647	0.286	0.696
Ether extract, %	82.58	83.39	82.12	82.59	1.52	0.947	0.855	0.913

Note. *n* = 8. SEM = standard error of mean. CON = control diet; DBSP = dried bamboo shoot powder. ^a,b^ Different superscript letters (*p* < 0.05).

**Table 6:** Effects of partial replacement of corn with dried bamboo shoot powder on serum biochemical indices in growing–finishing pigs.

Item	DBSP Replacement Level (% of Diet)				SEM	*p*-Value		
	0 (CON)	1.00	2.00	3.00		Treatment	Linear	Quadratic
ALT, U/L	64.48	66.39	65.31	65.12	1.20	0.727	0.878	0.390
AST, U/L	91.48	91.33	91.93	95.63	2.50	0.578	0.252	0.447
ALP, U/L	120.39	117.61	120.01	122.39	2.36	0.564	0.431	0.283
TP, g/L	70.87	70.73	70.83	71.10	0.87	0.992	0.840	0.817
ALB, g/L	34.94	33.00	33.59	34.11	0.73	0.305	0.567	0.104
GLU, mmol/L	4.02	3.95	3.97	4.17	0.25	0.927	0.694	0.591
BUN, mmol/L	7.43	7.71	7.19	7.90	0.34	0.481	0.560	0.529
TC, mmol/L	3.15	2.91	3.10	3.25	0.20	0.657	0.583	0.318
HDL-c, mmol/L	1.04	0.70	0.92	1.22	0.22	0.416	0.445	0.157
LDL-c, mmol/L	1.45	1.52	1.45	1.54	0.11	0.898	0.709	0.931

Note: Values are expressed as means, *n* = 8. SEM = standard error of the mean. ALT = alanine aminotransferase; AST = aspartate aminotransferase; ALP = alkaline phosphatase; TP = total protein; ALB = albumin; GLU = glucose; BUN = blood urea nitrogen; TC = total cholesterol; HDL-c = high-density lipoprotein cholesterol; LDL-c = low-density lipoprotein cholesterol.

**Table 7:** Effects of partial replacement of corn with dried bamboo shoot powder on serum immune and inflammatory indices in growing–finishing pigs.

Item	DBSP Replacement Level (% of Diet)				SEM	*p*-Value		
	0 (CON)	1.00	2.00	3.00		Treatment	Linear	Quadratic
IgA, g/L	1.66	1.68	1.54	1.79	0.31	0.951	0.861	0.698
IgG, g/L	9.84	9.51	9.48	9.54	0.61	0.972	0.735	0.749
IgM, g/L	1.34	1.47	1.73	1.69	0.29	0.751	0.319	0.774
TNF-α, ng/L	90.36	89.36	89.77	91.14	1.88	0.915	0.745	0.532
IL-6, ng/L	97.96	97.83	98.60	98.22	2.54	0.997	0.894	0.962
IL-10, ng/L	29.78	31.02	30.67	29.99	0.93	0.767	0.947	0.314

Note: Values are expressed as means, *n* = 8. SEM = standard error of the mean. IgA = immunoglobulin A; IgG = immunoglobulin G; IgM = immunoglobulin M; TNF-α = tumor necrosis factor-alpha; IL-6 = interleukin-6; IL-10 = interleukin-10.

**Table 8:** Effects of partial replacement of corn with dried bamboo shoot powder on serum antioxidant indices in growing–finishing pigs.

Item	DBSP Replacement Level (% of Diet)				SEM	*p*-Value		
	0 (CON)	1.00	2.00	3.00		Treatment	Linear	Quadratic
T-AOC, U/mL	10.99	10.35	10.24	10.50	0.35	0.455	0.317	0.211
SOD, U/mL	64.26	63.02	62.43	61.03	1.18	0.298	0.062	0.949
CAT, U/mL	45.44	44.93	42.91	45.52	1.31	0.467	0.760	0.243
GSH-Px, U/mL	642.94	634.83	626.06	650.85	31.78	0.952	0.917	0.609
MDA, μmol/L	4.70	5.03	5.24	4.38	0.52	0.668	0.752	0.262

Note: Values are expressed as means, *n* = 8. SEM = standard error of the mean. Treatment represents the overall effect of dietary treatment. Linear and quadratic responses were analyzed using orthogonal polynomial contrasts. DBSP = dried bamboo shoot powder.

**Table 9:** Effects of partial replacement of corn with dried bamboo shoot powder on colonic short-chain fatty acid concentrations in growing–finishing pigs.

Item, μmol/g	DBSP Replacement Level (% of Diet)				SEM	*p*-Value		
	0 (CON)	1.00	2.00	3.00		Treatment	Linear	Quadratic
Acetate	79.49	78.16	83.48	83.70	1.75	0.075	0.030	0.662
Propionate	18.96	18.63	21.95	20.34	1.60	0.459	0.308	0.692
Isobutyrate	18.30	17.32	18.88	19.31	0.97	0.512	0.301	0.472
Butyrate	22.05	19.61	19.96	20.12	0.99	0.321	0.229	0.202
Isovalerate	5.33	4.98	5.20	4.91	0.56	0.947	0.683	0.966
Valerate	6.53	5.91	7.30	6.22	0.73	0.578	0.894	0.752
Total SCFAs	150.66 ^ab^	144.62 ^b^	156.77 ^a^	154.59 ^ab^	2.69	0.019	0.057	0.479

Note. *n* = 8. SEM = standard error of mean. CON = control diet; DBSP = dried bamboo shoot powder. ^a,b^ Different superscript letters (*p* < 0.05).

**Table 10:** Effects of partial replacement of corn with dried bamboo shoot powder on carcass traits in growing–finishing pigs.

Item	DBSP Replacement Level (% of Diet)				SEM	*p*-Value		
	0 (CON)	1.00	2.00	3.00		Treatment	Linear	Quadratic
Carcass weight, kg	76.72	77.07	77.32	78.16	0.44	0.144	0.027	0.577
Carcass length, cm	107.06	107.55	106.08	108.01	0.94	0.523	0.745	0.451
Dressing percentage, %	63.20	64.14	63.89	64.28	0.32	0.096	0.042	0.385
Average backfat depth, mm	29.40	28.47	27.19	26.89	1.10	0.355	0.083	0.777

Note: Values are expressed as means, *n* = 8. SEM = standard error of the mean; DBSP = dried bamboo shoot powder. Treatment represents the overall effect of dietary treatment. Linear and quadratic responses were analyzed using orthogonal polynomial contrasts.

**Table 11:** Effects of partial replacement of corn with dried bamboo shoot powder on meat quality in growing–finishing pigs.

Item	DBSP Replacement Level (% of Diet)				SEM	*p*-Value		
	0 (CON)	1.00	2.00	3.00		Treatment	Linear	Quadratic
pH45 min	6.85	7.07	6.89	7.25	0.23	0.585	0.323	0.771
pH24 h	5.96	5.91	6.00	5.88	0.21	0.979	0.867	0.878
L*	42.71	41.76	38.99	41.24	1.22	0.198	0.201	0.202
a*	4.92	4.93	4.99	4.99	0.19	0.990	0.762	0.976
b*	7.24	7.02	7.22	7.23	0.18	0.792	0.840	0.529
Drip loss, %	1.90	1.99	2.00	1.93	0.21	0.984	0.912	0.705
Cooking loss, %	34.06	33.95	33.27	34.68	0.50	0.271	0.599	0.135
Shear force, N	9.19	9.07	9.24	9.79	0.35	0.493	0.222	0.350

Note: Values are expressed as means, *n* = 8. SEM = standard error of the mean; L*, a*, and b* represent lightness, redness, and yellowness, respectively; DBSP = dried bamboo shoot powder. Treatment represents the overall effect of dietary treatment. Linear and quadratic responses were analyzed using orthogonal polynomial contrasts.

## Data Availability

The original contributions presented in this study are included in the article. Further inquiries can be directed to the corresponding author(s).
